# Gelucire Based *In Situ* Gelling Emulsions: A Potential Carrier for Sustained Stomach Specific Delivery of Gastric Irritant Drugs

**DOI:** 10.1155/2013/436932

**Published:** 2013-11-10

**Authors:** Ashwin Saxena, Arun K. Mishra, Navneet Verma, Shiv S. Bhattacharya, Amitava Ghosh, Anurag Verma, Jayanta K. Pandit

**Affiliations:** ^1^Department of Pharmaceutics, College of Pharmacy, IFTM, Moradabad Uttar Pradesh 244001, India; ^2^Bengal College of Pharmaceutical Science and Research, Durgapur, West Bengal, India; ^3^Department of Pharmaceutics, Institute of Technology, Banaras Hindu University, Varanasi, India

## Abstract

Non steroidal anti-inflammatory drugs (NSAIDs) are commonly prescribed medications to the geriatric patients for the treatment of arthritis and other painful disorders. The major side effects of NSAIDs are related to their effects on the stomach and bowels. The present study concerns assessment of the potential of liquid *in situ* gelling emulsion formulations (emulgels) as patient compliant stomach specific sustained release carrier for the delivery of highly gastric irritant drug, Piroxicam. Emulgels were prepared, without using any emulgent, by mixing different concentrations of molten Gelucire 39/01 with low viscosity sodium alginate solution prepared in deionized water at 50°C. CaCO_3_ was used as buoyancy imparting as well as crosslinking agent. Emulgels so prepared were homogenous, physically stable, and rapidly formed into buoyant gelled mass when exposed to simulated gastric fluid (SGF, pH 1.2). Drug release studies carried out in SGF revealed significant retardation (*P* < 0.05) of Piroxicam release from emulgels compared to conventional *in situ* gelling formulations prepared without Gelucire 39/01. Pharmacodynamic studies carried out in *albino rats* revealed significantly increased analgesic/anti-inflammatory response from *in situ* emulgels compared to conventional *in situ* gelling formulations. Further, *in vivo* toxicity studies carried out in *albino rats* revealed no signs of gastric ulceration upon prolonged dosing.

## 1. Introduction

Development of sustained-release liquid oral dosage forms bearing gastric irritant nonsteroidal anti-inflammatory drugs (NSAID's) is beneficial for optimal therapy with respect to the efficacy, safety, and patient compliance, specifically for geriatric patients. Oral liquid dosage forms are more palatable and offer better patient compliance than tablets and capsules but often suffer from quick gastrointestinal transit. This could be a serious problem for the drugs with absorption window in the stomach. The gastric retention of oral liquid formulations containing such drugs could be successfully augmented through a strategy of liquid *in situ* gelling systems. These polymeric gel formulations respond to chemical or physical signals, including pH, metabolite, ionic factor, or temperature [1–4]. The polymers investigated principally for this purpose so far include alginates, gellan, pectin, chitosan, and so forth. Although *in situ* gelling systems possess a number of advantages in terms of biocompatibility and biodegradability, these systems are too fragile and do not have mechanical strength to hold the entrapped drug(s) in many cases. Combining hydrophilic polymers with lipid materials has been utilized in the past to solve the problem of fast drug release from hydrophilic matrices [7–12]. Upon the literature survey, it was observed that there is lack of studies reporting combination of lipids with hydrophilic polymers to develop liquid *in situ* gelling systems. Engstrom et al investigated the utility of the polar insoluble, but swelling, lipid monoolein which forms a highly ordered cubic phase in excess water, for the sustained release of different types of drugs [7]. The lipid system formed in this way acts similar to *in situ* activated gel-forming polymer systems and can be used for the application for a wide variety of drugs. Jauhari and Dash developed an *in situ* gelling drug delivery system consisting of chitosan and glyceryl monooleate in 0.33 M citric acid containing paclitaxel for sustained and targeted drug delivery for chemotherapy to mucin producing cancerous cells. The amount of paclitaxel transported across cell lines was much lower in case of the gel as compared with paclitaxel in solution indicating that this gel delivery system can be used to sustain the release of paclitaxel [12].

In the present investigation, an attempt has been made to develop *in situ* gelling emulgels using Gelucire 39/01 as lipid phase and low viscosity sodium alginate solution in deionized water as aqueous phase of emulsion. Gelucires are glycerides and polyglycerides of fatty acids of vegetable origin. Their amphiphilic nature is attributed to the long hydrocarbon chain and the alcohol moieties that make these bases suitable as a lipid carrier for both hydrophilic and lipophilic drugs. The terminology used for identification of Gelucires described their properties; the first number corresponds with the melting point of the material, on a scale of 33 to 65°C. The second number in the terminology represented the HLB value on scale extending from 0 to approximately 20. Grades with higher HLB values contain high portions of hydrophilic fractions (the more polar polyethylene glycol esters) while grades with lower HLB values contain higher portions of lipophilic fractions (particularly glycerides). The reason for selecting Gelucire 39/01 as lipid phase was attributed to its low susceptibility to oxidation, whereas conventional lipids generally suffer from the problem of rancidity [13]. Further, Gelucire 39/01 (glyceryl esters of fatty acids also known as hard fat) is official in the United States Pharmacopoeia (USP-NF), European Pharmacopoeia (EP) and included in IIG (inactive ingredients in FDA approved list). Like Gelucire 39/01, low viscosity sodium alginate is also nontoxic, biocompatible, and biodegradable and are included in a group of compounds that are generally regarded as safe (GRAS) by the FDA when given orally. Moreover, low viscosity sodium alginate promptly forms emulsion with Gelucire 39/01 without the need of any external surfactant.

Piroxicam is frequently used NSAID for long term treatment of chronic diseased conditions like osteoarthritis, rheumatoid arthritis, and so forth to improve patient's quality of life and ability to function. Like all NSAIDs, Piroxicam is also associated with elevated risk of gastrointestinal toxicity such as ulcers and bleeding. The risk may be higher for the people who are older in age, have poor health, or drink large amount of alcohol [14]. Oral delivery of such drugs through drug delivery carrier like *in situ* gelling emulsion gels not only prevents the direct contact of the drug with gastric mucosa but also sustains the release of embedded drug leading to better treatment efficacy, safety, and patient compliance.

## 2. Material and Methods

### 2.1. Materials

Piroxicam was gifted by Ranbaxy Laboratories Ltd. (New Delhi, India). Sodium alginate (viscosity determined by Brookfield viscometer = 210 cps of 2% aqueous solution) was purchased from Sigma-Aldrich Company (St. Louis, USA). Gelucire 39/01 (waxy solid, melting point 39°C, HLB = 01, acid value < 0.2, iodine value < 2) was a gift from Gattefosse (St Priest, Cedex, France). Deionized water used in the formulations was of HPLC grade (Merck) and all other chemicals used were of analytical grade.

### 2.2. Methods

#### 2.2.1. Drug-Excipient Interactions

While designing any drug delivery system, it is imperative to consider the compatibility of drug and polymers used within the system. Therefore, it is necessary to confirm that the drug is not interacting with polymers under experimental conditions and shelf life. For the present study, the drug-polymer interaction studies were performed using differential scanning calorimetry (DSC) and Fourier Transform Infrared Spectroscopy (FTIR). For DSC studies, approximately 5 mg samples were placed on a standard aluminium pan and heated at a rate of 10°C/min using a DSC facility (Universal V4.5A TA instrument). FTIR spectra were recorded for pure polymers, drug and, drug-loaded dried *in situ* gelling formulations using a FT-IR facility (Shimadzu, model 8400S). The samples were prepared in KBr disks (2 mg sample in 200 mg KBr). For FTIR analysis, the scanning range was 400–4000 cm^−1^ and the resolution was 2 cm^−1^.

#### 2.2.2. Preparation of *In Situ* Emulgels

*In situ* gelling emulsion formulations were prepared with sodium alginate and Gelucire 39/01 (Table 1). Emulsions were prepared by mixing with the help of a mechanical stirrer at about 500 rpm, sodium alginate dissolved in HPLC grade water (maintained at 500°C) with different concentrations of molten Gelucire 39/01 at 50°C. Emulsions were formed rapidly without the need of surfactants. Appropriate amount of CaCO_3_ and model drug Piroxicam were added to the emulgels at around 40°C. The total volume of all the formulations was 10 mL.

Conventional *in situ* gelling formulations were also prepared in the same manner but without Gelucire 39/01.

#### 2.2.3. Measurement of Rheological Properties of Emulsions

The viscosity of emulsions (drug-free) prepared in HPLC grade water was determined at 37 ± 1°C with Brookfield cone and plate rheometer with cone angle 0.8° (DV-III ULTRA) using spindle cp 40. A typical run comprised of changing the angular velocity from 0.5 to 100 rpm at a controlled ramp speed. After 6 seconds at 0.5 rpm, the velocity was increased to 100 rpm with similar wait at each speed. The hierarchy of angular velocity was reversed (100 rpm to 0.5 rpm) with similar wait of 6 seconds [15]. The average of two readings was used to calculate the viscosity. Evaluations were conducted in triplicate.

#### 2.2.4. *In Vitro* Gelation Study

The gelation studies were carried out in gelation cells, fabricated locally using Teflon. The cells were cylindrical reservoirs capable of holding 10 mL of simulated gastric fluid as gelation solution (SGF, pH 1.2). Within the cells located at the bottom is a transparent plastic cup to hold the gel sample in place after its formation. Two milliliters of the formulation was carefully placed into the cavity of the cup using a micropipette, and 6 mL of the gelation solution was added slowly and the rate of gelation was detected by visual examination [15].

#### 2.2.5. *In Vitro* Drug Release and Buoyancy Studies

*In vitro* buoyancy and drug release studies were carried out using a USP XXIII dissolution apparatus-II (Electrolab, Mumbai, India) with 900 mL of SGF (pH 1.2) at 37 ± 2°C with a paddle speed of 50 rpm. For determination of buoyancy lag time, a measured sample of 10 mL placed on a Petri dish was put into the dissolution medium. The time taken by the gelled mass to reach to the top of the dissolution medium (onset of floating) and the duration of floating were noted [15]. For determination of drug release studies, 10 mL of the formulation was transferred as above in the dissolution vessel. At a preidentified time interval, an aliquot was removed and replenished with fresh medium. The samples were assayed for Piroxicam at 354 nm using spectrophotometer (Shimadzu 1800, Japan) after suitable dilution. A concurrent dissolution was performed with preparation devoid of drug to record the interference from excipients, if any. All the studies were conducted in triplicate and the average was recorded.

### 2.3. Mechanism of Drug Release

In order to describe the kinetics of Piroxicam release from hydrodynamically balanced capsule formulations, various equations were used, such as the zero-order rate equation, which describes the systems where the release rate is independent of the concentration of the dissolved species. The first-order equation describes the release from systems where dissolution rate is dependent on the concentration of the dissolving species [16]. *In vitro* data were also fitted to Higuchi's square root model [17] which describes the release from systems where the solid drug is dispersed in an insoluble matrix and the rate of drug release is related to the rate of drug diffusion. Release of a drug from an insoluble matrix is the square root of a time-dependent process based on Fickian diffusion:

(1) $$\begin{array}{c} Q_{t}=kHt^{1/2}, \end{array}$$

where *Q*_*t*_ is the amount of drug released in time *t*  and *kH* is the release rate constant for the Higuchi model. Under some experimental situations, the release mechanism deviates from Fick's equation, following an anomalous behavior (non-Fickian release). In these cases, a more generic equation can be used. Korsmeyer et al. [18] developed a simple, semi-empirical model, relating exponentially the drug release to the elapsed time. Consider

(2) $$\begin{array}{c} Mt/M_{\infty }=\;kt^{n}, \end{array}$$

where *Mt*/*M∞* is the fraction of drug released at time *t*; *k* is a constant reflecting the design variables of the system and *n*, the release exponent, is a parameter that depends on the release mechanism and is thus used to characterize it. If the *n*  value is 0.5 or less, the release mechanism follows Fickian diffusion, and values 0.5 < *n* < 1 for mass transfer follow a non-Fickian model (anomalous transport). Drug release follows zero-order drug release and case-II transport if the *n* value is 1. For the values of *n* higher than 1, the mechanism of drug release is regarded as super case-II transport. This model is used to analyze the release from pharmaceutical polymeric dosage forms when the release mechanism is not well known or when more than one type of release phenomena is involved.

### 2.4. Animal Studies

Male *albino rats* (150–200 gm) purchased from the CDRI (Lucknow, India) was used in the animal studies. The animals were kept under standard laboratory conditions, at 25 ± 10°C and 50 ± 5% relative humidity with a 12 h light/dark cycle. The animals were housed in polypropylene cages, with free access to a standard laboratory diet and water. All surgical and experimental procedures were reviewed and approved by the Animal and Ethics Review Committee, College of Pharmacy, IFTM, Moradabad, India.

#### 2.4.1. *In Situ* Gel Formation in *Rat* Stomach

The study was to examine the influence of the gastric environment on the formation of the gel structure after administering a dose of the tested formulae. One mL of solutions of formulations E3 and E5 was orally administered to the experimental animals (*n* = 3). The stomach was excised immediately after administration, after 30 min, and after 60 min. The formed gels were removed and weighed after removing the surface dirt.

### 2.5. Pharmacodynamic Studies 

#### 2.5.1. Analgesic Activity

*Tail Immersion Method.* The prescreened animals (reaction time 6-7 sec) were divided into five groups with each group having three animals. One group served as the control was treated with 1 ml/kg of saline orally. The second group that served as standard was given Piroxicam suspension (1 mg/kg) by the same route. The remaining groups that served as experimental group were treated with the formulation (E3 and E5) at a dose of 1 mg/kg orally by intubation. The tail flick latency was assessed by the analgesiometer (Inco, India). The strength of the current passing through the naked nichrome wire was kept constant at 6 Amps. The distance between the heat source and the tail skin was 1.5 cm [19]. The site of application of the radiant heat in the tail was maintained at 2.5 cm, measured from the root of the tail. The cut-off reaction time was fixed at 10 sec to avoid tissue damage. The initial reading was taken immediately before administration of test and standard drugs and then by 30, 60, 90, and 120 min after the administration.

#### 2.5.2. Anti-Inflammatory Activity

*Rat Paw Edema Method. *The animals were divided into five groups (*n* = 3 in each group). Acute inflammation was produced by subplantar injection of 0.1 mL of 1% suspension of carrageenan with 2% gum acacia in normal saline, in the right hind paw of the rats, one hour after oral administration of normal saline (control), Piroxicam suspension (standard), and formulations E3 and E5 (treatment). The paw volume was measured plethysmometrically (Ugo Basile, Italy) at “1” hour interval up to “6” hours after the carrageenan injection and volume of edema and the percentage of anti-inflammatory activity were calculated [20].

*In Vivo Gastric Toxicity Studies. *In order to study the ulcerogenic potential of *in situ* emulgels, *albino rats* were orally administered one mL *in situ* emulgels (1 mg drug/mL) for consecutive four days and stomachs of the *animals* were removed and examined histopathologically. The tissues were fixed in 10% buffered formalin and were processed using a tissue processor. The processed tissues were embedded in paraffin blocks and about 5 **μ**m thick sections were cut using a rotary microtome. These sections were stained with hematoxylin and eosin. The slides were examined microscopically for ulcers. The sections under microscope were photographed at original magnification ×100.

### 2.6. Statistical Analysis

All the data were analyzed by Student's *t*-test and one-way ANOVA to determine statistical difference in the results. A probability value *P* < 0.05 was considered statistically significant. The software used was SigmaPlot 11 (Systat Software Inc.).

## 3. Result and Discussion

Better medical treatments do not always require a stronger medicine. The effectiveness of medicinal agents depends on the type of dosage form in which they are incorporated and method of administration, so treatments can often be improved by finding optimal drug formulations or delivery systems. *In situ* emulgels are liquid at room temperature, but they undergo gelation when in contact with body fluids due to cation induced gelation and are expected to release the loaded drug, Piroxicam, in a sustained manner. Piroxicam loaded *in situ* emulgels are expected to remain in intimate contact with the absorbing site, thus maximizing both rate and extent of drug absorption, without causing significant gastric discomfort. These *in situ* gel preparations can be easily formulated in bulk and these formulations give homogeneity of drug distribution when compared to other conventional suspensions.

### 3.1. Drug-Excipient Interactions

Drug-excipients interactions are one of the most important characteristics that regulate the drug release and pattern from the formulations and its stability in the formulation. Drug-excipient interactions were studied at the very outset before the beginning of the development of formulations. Various methods such as DSC and FTIR spectra. are frequently used to study drug-excipient interaction. The FTIR spectrum can accurately clarify drug-excipient interactions at the various functional groups between the drug and excipient molecules.

**Figure 1:**
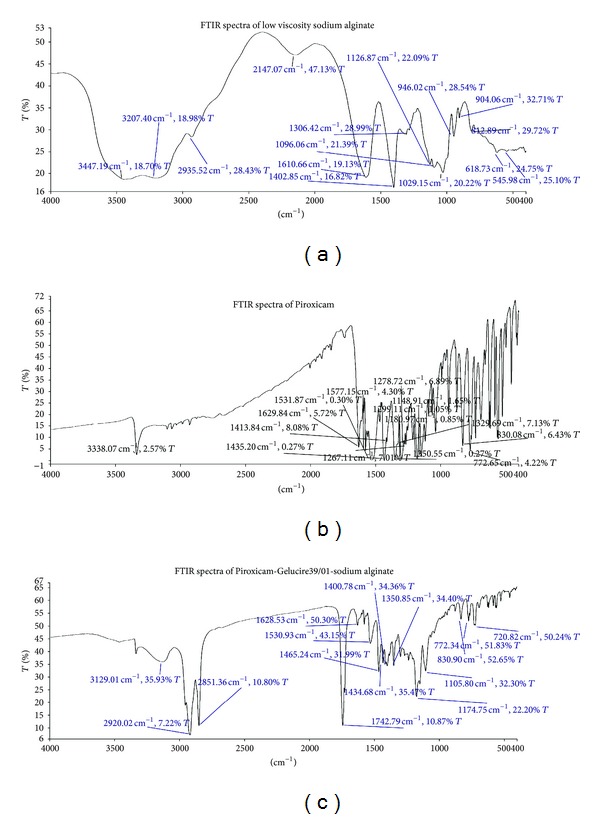
FTIR spectra of low viscosity sodium alginate, Piroxicam and dried emulgel (E3, *in situ* gelling emulsion formulation after gelation).

The FTIR spectra of pure Piroxicam represent (Figure 1) absorption band at 3338.07 cm^−1^ which indicate that the drug is in the cubic polymorphic form. Absorption band at 772.65, 1148.91, 1350.55, 1435, and 1629.84 cm^−1^ corresponds to stretching of ortho-di-substituted phenyl, stretching of –SO_2_-N-group, stretching of symmetric methyl group, stretching of asymmetric methyl group, and stretching of amide carbonyl, respectively. The FTIR spectra of sodium alginate show that (Figure 1) peaks at 1610.66 and 1402.85 cm^−1^ are due to asymmetric and symmetric stretching of carboxylate groups. The absorption band at 2925.52 cm is attributed to –CH_2_ group. The FTIR spectra (Figure 1) of dried emulgels showed that there was no major shifting of functional peaks and no overlapping of characteristic peaks and also there was no appearance of new peaks. This indicates that the dried emulgel spectra were only the summation of spectra of Piroxicam and individual excipient. The DSC analysis was carried out over 50–250°C at 10°C/min, using duplicate samples of 5 mg in crimped aluminum pans. Indium samples were used to calibrate the DSC instruments.

**Figure 2:**
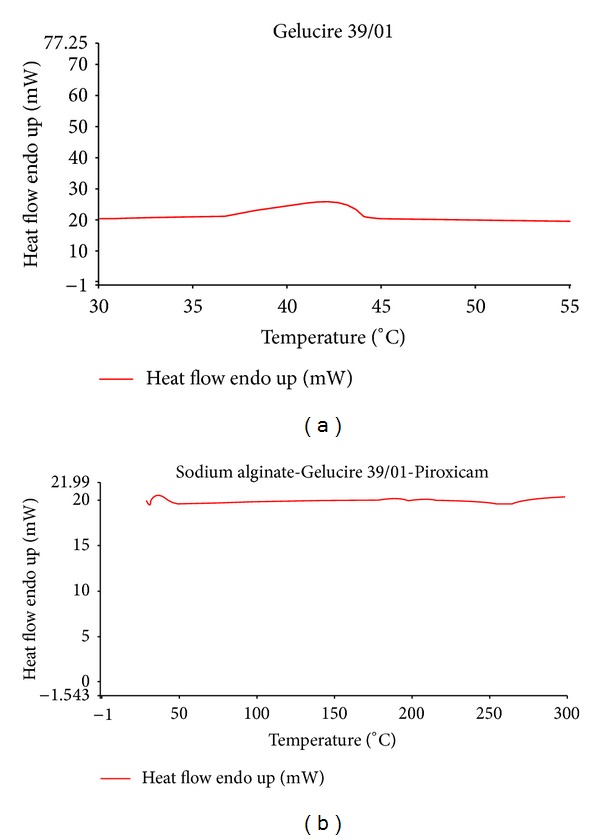
DSC thermograms of Gelucire 39/01 and dried emulgel (E3, *in situ* gelling emulsion formulation after gelation).

The DSC thermogram of Gelucire 39/01 exhibited a broad endothermic peak with melting onset and end point ranges from at around 37°C to 44°C (Figure 2). This broad range could be attributed to the melting of lipid fractions with different melting points in the Gelucire 39/01. The DSC thermogram of Piroxicam showed a sharp peak endothermic peak at 204.8°C corresponding to the melting point of the drug (data not shown). The DSC thermogram of dried emulgel (E3) showed that a broad peak ranges from 35°C to 45°C, whereas peak for the drug was completely absent. This may suggest that the drug is homogenously mixed or dissolved in the Gelucire matrix.

Since Gelucire 39/01 is a lipid and consists of a mixture of short, medium, and long chain fatty acids of carbon chain length C-12 to C-18 (myristic acid (C-14), palmitic acid (C-16), stearic acid (C-18), caproic acid, capric acid (C-10), lauric acid (C-12), caprylic acid (C-8), linolie acid, and oleic acid) [13], it is desirable to gain knowledge of effect of drug incorporation on the structure of the lipid, as this may affect the release pattern of drugs from the lipid matrix. For this purpose Piroxicam: Gelucire 39/01 (5 : 95, 10 : 90, 15 : 75 and 30 : 70) solid dispersions were prepared by melt granulation techniques. It was observed that as the concentration of drug in the Gelucire matrix increases, the consistency of solid dispersion changes from solid (5 : 95, 10 : 90) to semisolid (15 : 75) to viscous solution (30 : 70, color changes to yellow from white but no degradation of drug) at room temperature (30°C). This change in consistency could be attributed to the decrease in higher melting point fraction of fatty acids as drug concentration increases above 20 part drug to Gelucire 39/01 level. Due to this, only 5, 10, and 15 part drug to Gelucire 39/01 solid dispersions were considered for thermal studies.

**Figure 3:**
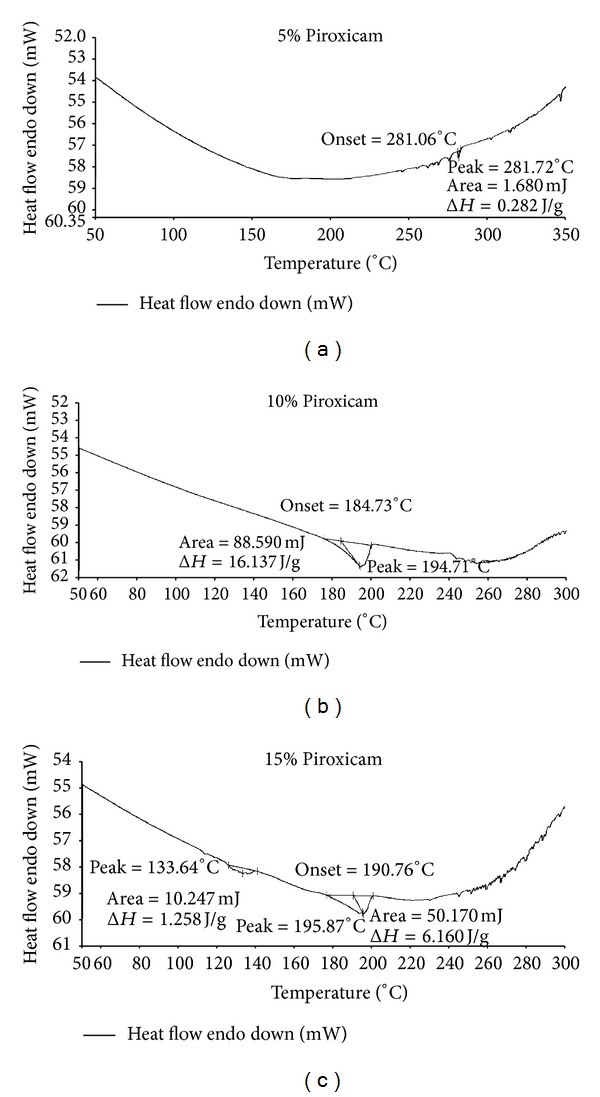
DSC thermograms of Piroxicam: Gelucire 39/01 solid dispersion containing 5, 10, and 15% Piroxicam.

Thermogram obtained (Figure 3) from 5, 10, and 15 part drug to Gelucire ratio revealed that at 5 part drug Gelucire level; Piroxicam seems to be homogenously mixed in Gelucire matrix as there was no peak due to the drug. In case of 10 and 15 parts drug to Gelucire ratio, broad endothermic peaks due to melting of drug appeared at lower temperature, that is, at 194°C. This could be attributed to the molecular dispersion of the drug in the lipidic Gelucire matrix, which has resulted in high specific surface area and thus melting at lower temperature.

### 3.2. Preparation of *In Situ* Gelling Formulations

The preparation of *in situ* gelling emulgels is simple, reliable, and reproducible involving dispersion of Piroxicam in emulsion (sodium alginate and Gelucire based emulsions). The two main prerequisites of *in situ* gelling systems are optimum viscosity and gelling capacity (speed and extent of gelation). The formulation should have an optimum viscosity that will allow easy swallowing as a liquid, which then undergoes a rapid sol-gel transition due to ionic interaction [15]. The various physical parameters of prepared formulations are summarized in Table 2. 

As the *in situ* gelling formulations (E1–E4; E5 and E6) came in contact with dissolution medium, CaCO_3_ effervesces to produce CO_2_ and simultaneously released Ca^++^ ions. Released Ca^++^ ions interacted with COO^−^ on alginate network causing formation of strong aggregation of pairs of helices (gelled network), whereas the released carbon dioxide is entrapped in the gel network of the formulations, and gel rises to the surface of the dissolution medium (*in vitro*) or the stomach fluid (*in vivo*). This low density structure had consistency of a soft, palpable swollen lipid-hydrocolloid matrix which remained buoyant on SGF, without disintegrating, throughout the period of experiment. However a lag time in the buoyancy was observed. *In situ* emulgels (E1, E2, E4, and E4) exhibited 69 to 87 seconds of buoyancy lag time, whereas conventional *in situ* gelling formulations (E5 and E6) 67 and 71 seconds, respectively. It was observed that, the viscosity of emulgels was relatively higher than conventional *in situ* gelling formulations and was found to be dependent upon Gelucire 39/01 as well as drug concentration. Further, Gelucire 39/01, being highly lipophilic excipient, confers a certain degree of hydrophobicity to the emulgels which retarded the rapid penetration of dissolution fluid inside the emulsion structure, thus, increased buoyancy lag time.

### 3.3. *In Situ* Gel Formation in *Rat *Stomach

**Figure 4:**
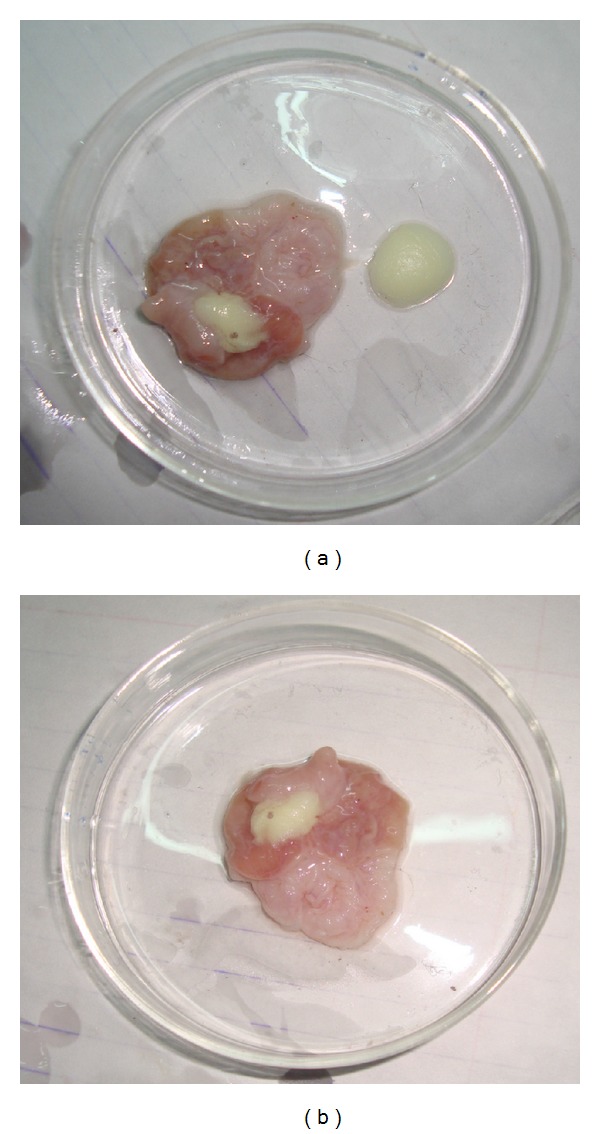
Photographs showing formation of gel in rats stomach after administration of 1 mL of *in situ* emulgel formulation E3.

**Figure 5:**
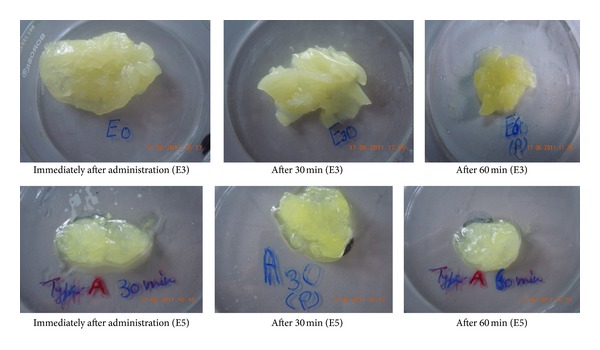
Photograph showing formation of gel in rat stomach (E3 and E5).

The study was to examine the influence of the gastric environment in the stomach of the *albino rats* on the formation of the gel structure after administering a dose of the tested formulae (E3 and E5). Visual observation of the stomach contents oral administration of 1 mL of formulations is depicted in Figures 4 and 5.

In case of emulsion formulation (E3), well-formed gels were observed in the stomach immediately after administration (Figure 4). At 30 and 60 min after administration, marked improvement in consistency of formed gels was observed (Figure 5). On the other hand, in case of alginate based *in situ* gelling formulation (E5), weak gel formation was observed in the stomach immediately after administration (Figure 5). Although the consistency of the gel improved after 30 and 60 min, respectively, these gels seemed to be weaker in consistency compared to E3.

### 3.4. *In Vitro* Drug Release Studies

**Figure 6:**
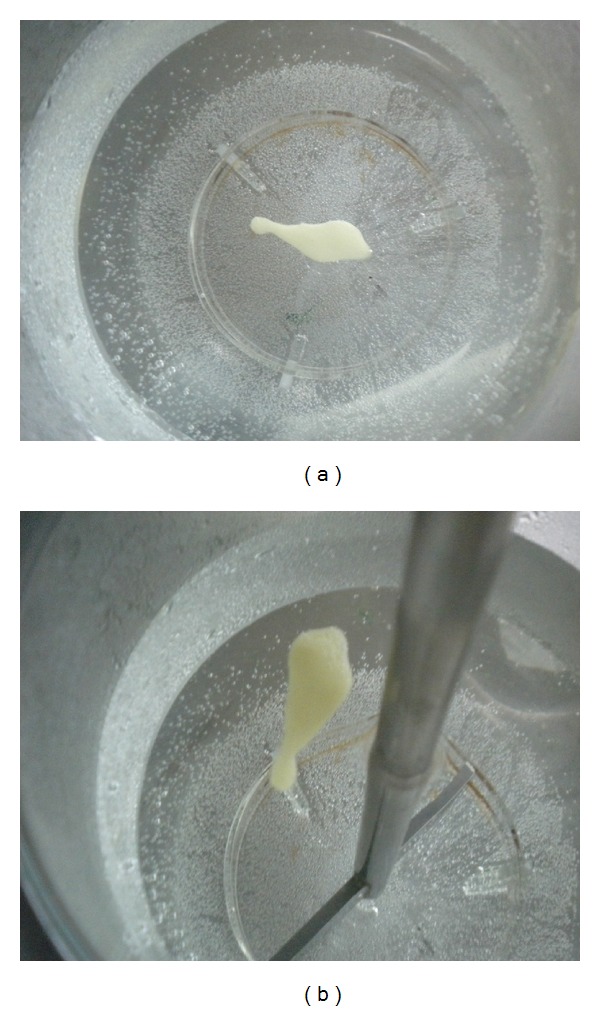
Photographs showing *in vitro* gelation and buoyancy of emulgels (E3).

**Figure 7:**
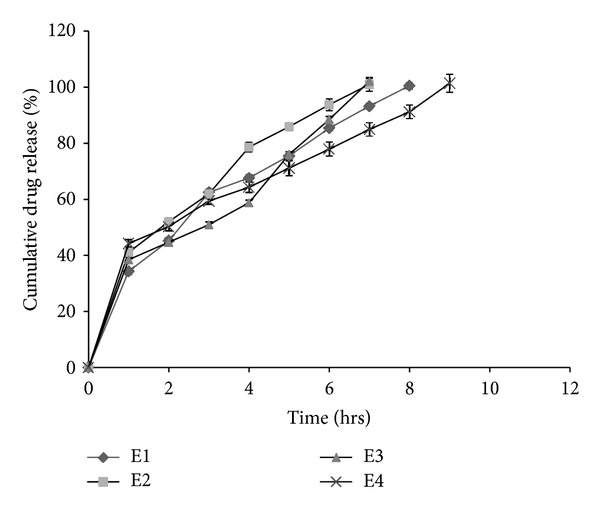
Cumulative drug release (%) from *in situ* emulgels.

Drug release studies were carried out in USP type II dissolution rate test apparatus using SGF (pH 1.2) as dissolution medium at 37 ± 0.5°C (Figure 6). *In situ* gelling emulgels are basically emulsions before coming into contact with acidic dissolution medium. When the *in situ* emulgel formulations come in contact with acidic dissolution medium, contained CaCO_3_ effervesces to release CO_2_ and Ca^++^. Free Ca^++^ ions then induce the gelation due to dimeric association of G block regions of sodium alginate [15]. At the same time, the lipid part of the emulsions, Gelucire, further increases the viscosity of the formulations that gives rise to highly viscous, thixotropic dispersions, with viscosities found to be dependent upon Gelucire 39/01 concentration. Significant retardation (*P* < 0.05) of Piroxicam release from *in situ* gelling emulgels was observed (Figure 7). The drug release decreased with increase in Gelucire 39/01 concentration (*P* < 0.05, E1 compared to E3) due to concomitant increase in viscosity of the formulations. Increasing the drug concentration from 25 mg to 50 mg (E4), it was expected that matrix of gel formed becomes more relaxed allowing easy penetration of dissolution fluid leading to enhanced drug diffusion; however, no significant (*P* < 0.05) influence over drug release was observed.

### 3.5. Conventional *In Situ* Gelling Formulations

Drug release from conventional *in situ* gelling formulations (E5 and E6) was too rapid with around 65% of the drug that was released within first hour in both cases. The release of drug from these gels (E5 and E6) was characterized by an initial phase of high release (burst effect). This may be due to the use of low viscosity sodium alginate, and formed gel may be weak or not sufficient to sustain the release of drug as evidenced froms Figure 5, which clearly demonstrate the difference in consistency of formed gels.

### 3.6. Mechanism of Drug Release

The *in vitro* release pattern of various formulations was analyzed by fitting the dissolution data into various kinetic models. Piroxicam release from *in situ* gelling emulgels showed linearity towards Higuchi square root model (0.9904, 0.9952, 0.9558, and 0.9809 for formulations E1, E2, E3, and E4), indicating the release mechanism to be diffusion based. Higuchi model describes the release from systems where the solid drug is dispersed in an insoluble matrix and the rate of drug release is related to the rate of drug diffusion [17]. The release exponent (*n*) of the formulations suggests (ranges from 0.37 to 0.50) that depending upon formulation variables, the Piroxicam release followed Fickian or quasi-Fickian mechanism. Formulations E5 and E6 also followed the Higuchi model with *n* values (0.28 and 0.41, resp.)indicating quasiFickian release pattern.

### 3.7. Pharmacodynamic Studies

Human volunteers are considered to be the ultimate model for *in vivo* studies of new formulations. However, to avoid unnecessary human testing, animal models are used initially during product development stage to tune the formulation to the desired specifications. The use of animal model provides some important advantages such as the possibility to perform early *in vivo* studies during preclinical drug development. These studies are cheaper and faster than human studies. Moreover, animal models are useful not only for preclinical screening tool but also for later mechanistic evaluations of finding obtained in human studies. After oral administration to *albino rats* (1 mg/kg Piroxicam), pharmacodynamic responses obtained from *in situ* emulgels (E3) were compared with that of control (normal saline), Piroxicam suspension, and conventional *in situ* gelling system (E5). Results are depicted in Tables 3 and 4.

Table 3 represented the results of comparison of analgesic effect at 1 mg/kg body weight from control, standard, and emulsion based *in situ* gelling formulations. Formulation E3 exhibited significant analgesic response (*P* < 0.05, E3 compared to standard) as analgesia began in 30 min and the peak effect was observed at the end of 90 min (*P* < 0.05, E3 compared to standard and E5), and after that, it begins to decline.

Table 4 represents the anti-inflammatory response obtained from control, standard, and floating *in situ* gelling formulations (E3 and E5). The observed responses were obtained by measuring change in the volume of inflamed paw, produced by injection of carrageenan using plethysmometer. Control group showed progressive inflammation up to six hours. With standard (Piroxicam suspension), there was *≈*36% inhibition of inflammation compared to control at the end of first hour and at the end of fifth hour it was *≈*44%. On the other hand, floating *in situ* gelling formulations (E3 and E5) exhibited *≈*31% inhibition at the end of first hour but at the end of five hours it was 62% (E3) and *≈*54% (E5), respectively, which were significantly different (*P* < 0.05) from standard and control. It was also observed that, although standard formulation showed better % inhibition of inflammation in the beginning due to relatively fast drug release, overall anti-inflammatory activity was more pronounced and extended with floating *in situ* emulgels (E3).

### 3.8. *In Vivo* Toxicity Studies

Piroxicam is a gastric irritant drug, and it can cause gastric ulceration upon long use [14]. Therefore, it becomes necessary to check the ulcerogenic potential of *in situ* emulgels. *Albino rats* (*n* = 3) were orally administered one mL of Piroxicam (1 mg/mL) suspension, conventional *in situ* gelling formulation and *in situ* emulgels for consecutive seven days and stomachs of the animals were removed and examined histopathologically.

**Figure 8:**
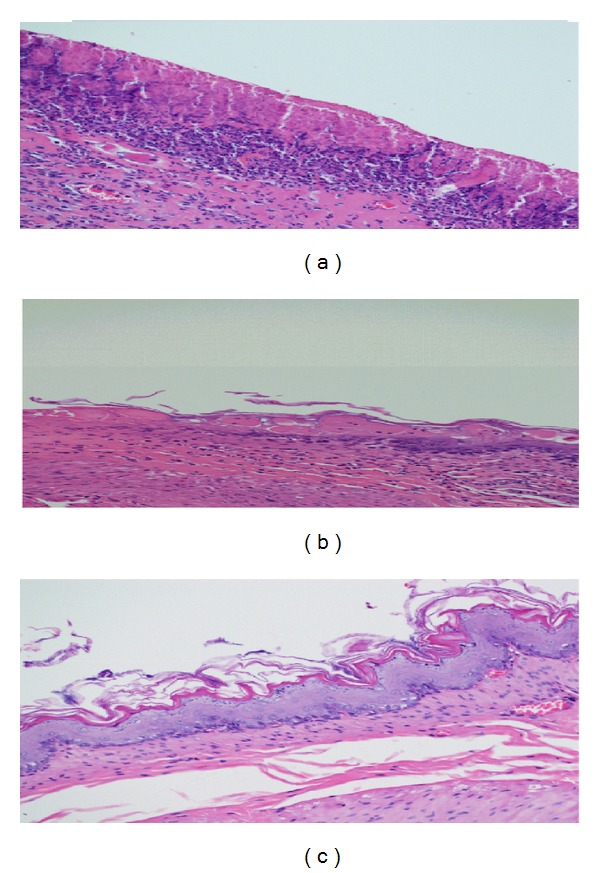
Stomach tissues of *albino rats* treated with Piroxicam suspension (1 mg/mL) for seven days showing gastric erosions appeared as red spots (a); *albino rats* treated with formulation E5 (1 mg/mL) for seven days also showing gastric erosions appeared as red spots (b); *albino rats* treated with E3 (1 mg/mL) for seven days showing no sign of gastric erosions.

Figure 8(a) shows histopathology of stomach of *albino rats* administered with Piroxicam suspension, which shows distorted glandular architecture with obvious signs of gastric erosions.  Figure 8(b) shows the histopathology of stomach of albino rats administered with formulation E5, which also shows distorted glandular architecture, but intensity of gastric erosions appeared to be reduced. Whereas no sign of erosions/ulcers was observed (Figure 8(c)) in the stomach tissues of animals administered with *in situ* emulgels (E3).

## 4. Conclusions 

In the presented investigation, *in situ* gelling emulgels capable of floating on SGF have been proposed as potential carrier for the sustained stomach specific delivery of gastric irritant drugs like NSAIDs, especially for geriatric patients. This study demonstrates that *in situ* gelling emulgels have the feasibility of forming gel both *in vitro* and *in vivo* and are capable of sustaining the release of model drug, Piroxicam, over a period of 8 to 10 hours. Furthermore, the analgesic and anti-inflammatory response obtained in experimental *albino rats* from *in situ* gelling emulgel (E4) showed significant improvement in both duration and intensity of responses compared to Piroxicam containing conventional *in situ* gelling formulation (E5) and suspension, without showing signs of significant gastric irritation. The method of preparation of investgated *in situ* emulgels is simple and amenable to easy scale up. In conclusion, it is suggested that *in situ* gelling emulgels may constitute a better option for sustained stomach specific delivery of gastric irritants drugs having absorption window in upper GIT, in terms of efficacy, patient compliance, and possibly improved safety profile especially for geriatric population.

## Figures and Tables

**Table 1:** Formulation composition of various *in situ* gelling formulations.

Formulation code	Sodium alginate (% w/v)	Gelucire 39/01 (mg)	CaCO_3_ (% w/v)	Piroxicam (mg)
E1	1.5	300	1	25
E2	1.5	200	1	25
E3	1.5	100	1	25
E4	1.5	100	1	50
E5	1.5		1	25
E6	1.5		1	50

**Table 2:** Absolute viscosities, floating lag time, and duration of floating of various formulations.

Formulation code	Absolute viscosity (cps)	Buoyancy lag time (seconds)	Duration of floating (hours)
E1	1240.1 ± 130.4	87 ± 8	24
E2	9521.2 ± 125.6	80 ± 5	24
E3	4512.4 ± 103.1	65 ± 4	24
E4	7012.3 ± 107.3	69 ± 5	24
E5	333.7 ± 11.5	67 ± 6	24
E6	375.1 ± 12.3	71 ± 5	24

**Table 3:** Analgesic response in *albino rats* obtained from Piroxicam containing *in situ* gelling formulations E3 and E5 compared to suspension formulation and control using tail immersion method.

Treatment	Dose	Analgesic response (mean time in seconds ± SEM)			
		30 min	60 min	90 min	120 min
Control (normal saline)	1.0 mL/kg	7.03 ± 0.44	7.34 ± 0.44	7.71 ± 0.25	7.32 ± 0.21
E5	1.0 mg/kg	17.66 ± 1.54	19.76 ± 0.26*	18.41 ± 0.47*	17.91 ± 0.45*
E3	1.0 mg/kg	22.51 ± 2.01	25.36 ± 0.21*	25.66 ± 0.29*	21.05 ± 0.33*
Standard (Piroxicam suspension)	1.0 mg/kg	16.95 ± 0.26*	17.41 ± 0.37*	18.3 ± 0.84*	17.81 ± 1.79*

Values are expressed as mean ± S.E.M. (*n* = 3); *P < 0.05 versus control.

**Table 4:** Anti-inflammatory response obtained from Piroxicam containing *in situ* gelling formulations (E3 and E5) compared to suspension formulation and control using carrageenan induced hind paw oedema method.

Treatment	Dose	Volume of mercury displaced (in mL.)					
		1 hr	2 hr	3 hr	4 hr	5 hr	6 hr
Control (normal saline)	1 mL/kg	0.42 ± 0.108	0.44 ± 0.102	0.58 ± 0.0131	0.72 ± 0.0103	0.90 ± 0.0147	0.92 ± 0.0134
Standard (Piroxicam suspension)	1 mg/kg	0.27 ± 0.009* (35.71%)	0.28 ± 0.008* (36.36%)	0.34 ± 0.012* (41.37%)	0.41 ± 0.012* (43.05%)	0.50 ± 0.018* (44.44%)	0.54 ± 0.016* (40.00%)
E3	1 mg/kg	0.29 ± 0.002 (30.95%)	0.29 ± 0.005 (34.09%)	0.28 ± 0.005* (51.71%)	0.30 ± 0.011* (58.33%)	0.34 ± 0.042* (62.22%)	0.40 ± 0.009 (55.55%)
E5	1 mg/kg	0.29 ± 0.002 (30.95%)	0.28 ± 0.009* (36.36%)	0.31 ± 0.005* (46.55%)	0.37 ± 0.005* (48.61%)	0.41 ± 0.009* (54.44%)	0.47 ± 0.01 (48.91%)

## References

[1] Miyazaki S, Kawasaki N, Kubo W, Endo K, Attwood D (2001). Comparison of in situ gelling formulations for the oral delivery of cimetidine. *International Journal of Pharmaceutics*.

[2] Kubo W, Miyazaki S, Attwood D (2003). Oral sustained delivery of paracetamol from *in situ*-gelling gellan and sodium alginate formulations. *International Journal of Pharmaceutics*.

[3] Prabaharan M, Mano JF (2006). Stimuli-responsive hydrogels based on polysaccharides incorporated with thermo-responsive polymers as novel biomaterials. *Macromolecular Bioscience*.

[4] Sophie R, Tomme V, Storm G, Wim Hennink E (2008). *In situ* gelling hydrogels for pharmaceutical and biomedical applications. *International Journal of Pharmaceutics*.

[5] El Maghraby GM, Elzayat EM, Alanazi FK (2012). Development of modified in situ gelling oral liquid sustained release formulation for dextromethorphan. *Drug Development and Industrial Pharmacy*.

[6] Ganguly S, Dash AK (2004). A novel *in situ* gel for sustained drug delivery and targeting. *International Journal of Pharmaceutics*.

[7] Engstrom S, Lindahl L, Wallin R, Engblom J (1992). A study of polar lipid drug carrier systems undergoing a thermoreversible lamellar-to-cubic phase transition. *International Journal of Pharmaceutics*.

[8] Mirghani A, Idkaidek NM, Salem MS, Najib NM (2000). Formulation and release behavior of diclofenac sodium in Compritol 888 matrix beads encapsulated in alginate. *Drug Development and Industrial Pharmacy*.

[9] Kim M-S, Park G-D, Jun S-W, Lee S, Park J-S, Hwang S-J (2005). Controlled release tamsulosin hydrochloride from alginate beads with waxy materials. *Journal of Pharmacy and Pharmacology*.

[10] Pongjanyakul T, Sungthongjeen S, Puttipipatkhachorn S (2006). Modulation of drug release from glyceryl palmitostearate-alginate beads via heat treatment. *International Journal of Pharmaceutics*.

[11] Al-Taani B, Khanfar MS, Salem MS, Sallam A (2010). Release behaviour of diclofenac sodium dispersed in Gelucire and encapsulated with alginate beads. *Journal of Microencapsulation*.

[12] Jauhari S, Dash AK (2006). A mucoadhesive *in situ* gel delivery system for paclitaxel. *AAPS PharmSciTech*.

[13] Sutananta W, Craig DQM, Newton JM (1995). An evaluation of the mechanisms of drug release from glyceride bases. *Journal of Pharmacy and Pharmacology*.

[14] Castellsague J, Riera-Guardia N, Calingaert B (2012). Individual NSAIDs and upper gastrointestinal complications: a systemic review and meta-analysis of observational studies (the SOS project). *Drug Safety*.

[15] Nagarwal RC, Kant S, Singh PN, Maiti P, Pandit JK (2009). In situ forming formulation: development, evaluation, and optimization using 3^3^ factorial design. *AAPS PharmSciTech*.

[16] Bourne DWA, Banker GS, Rhodes CT (2002). Pharmacokinetics. *Modern Pharmaceutics*.

[17] HIGUCHI T (1961). Rate of release of medicaments from ointment bases containing drugs in suspension. *Journal of Pharmaceutical Sciences*.

[18] Korsmeyer RW, Gurny R, Doelker E, Buri P, Peppas NA (1983). Mechanisms of solute release from porous hydrophilic polymers. *International Journal of Pharmaceutics*.

[19] Ghosh MN (1984). *Evaluation of Analgesic Agents, Fundamentals of Experimental Pharmacology*.

[20] Sheth UK, Dadkar NK, Kamt UG (1972). *Drugs Acting on CNS, Selected Topics in Experimental Pharmacology*.

